# Tumor Lysis Syndrome After mFOLFOX6 Administration for Ascending Colon Cancer

**DOI:** 10.7759/cureus.84896

**Published:** 2025-05-27

**Authors:** Yuta Kano, Tetsuhito Muranaka, Wataru Saito, Yusuke Honma, Daisuke Yokoyama, Yutaro Otsuka, Soichiro Matsuda, Yunosuke Takishin, Yasuyuki Kunieda

**Affiliations:** 1 Internal Medicine, Wakkanai City Hospital, Wakkanai, JPN

**Keywords:** ascending colon cancer, febuxostat, liver metastasis, modified folfox6 (mfolfox6) therapy, tumor lysis syndrome

## Abstract

Tumor lysis syndrome (TLS) is an oncologic emergency characterized by massive tumor cell lysis accompanied by the excessive release of large amounts of intracellular electrolytes and metabolites into the bloodstream. TLS is a potentially life-threatening condition that can lead to acute kidney injury, seizures, and sudden death due to arrhythmias. Therefore, prophylactic measures and prompt therapeutic intervention are essential for its management. TLS in solid tumors is extremely rare and has been documented only in a limited number of case reports. To our knowledge, three previous cases of TLS with colorectal cancer undergoing chemotherapy have been reported in the literature. We successfully treated a patient who developed TLS following modified FOLFOX6 (mFOLFOX6) therapy. The patient had ascending colon cancer with liver metastasis and right ureteral invasion and underwent colostomy before the initiation of mFOLFOX6. The presence of liver metastasis and elevated lactate dehydrogenase (LDH) levels on pre-treatment blood tests was considered a risk factor for the development of TLS. Therefore, blood tests were performed on day three after the initiation of mFOLFOX6 therapy, which revealed the onset of TLS. Prompt treatment with intravenous hydration, administration of diuretics, and oral febuxostat led to rapid improvement in serum uric acid, potassium levels, and renal function, allowing successful management without progression to a severe situation.

This case further emphasizes that early identification and treatment of TLS are critical for the prevention of irreversible organ damage. Although TLS does not present with specific clinical symptoms, this case highlights the importance of close monitoring through blood tests even after the initiation of chemotherapy in patients with solid tumors who are at risk for TLS. Particularly in tumors with known risk factors for TLS, it is essential to perform blood tests by the third day after the initiation of chemotherapy to assess for the development of TLS.

## Introduction

Tumor lysis syndrome (TLS) is a rare but serious complication in patients treated for solid tumors; it is characterized by metabolic abnormalities, such as hyperuricemia, hyperkalemia, hyperphosphatemia, and hypocalcemia [1]. The incidence of TLS in solid tumors has been reported to be less than 0.3% [2]. TLS can occur during or after chemotherapy, with a higher incidence observed in aggressive tumors that are relatively sensitive to chemotherapy, such as small cell lung cancer or breast cancer [2,3]. Mortality of TLS in solid tumors can be as high as 35% [4], which is surprisingly higher than TLS mortality in haematological malignancies (1.9%) [5]. According to the Cairo-Bishop classification [6], TLS is divided into laboratory TLS and clinical TLS based on specific biochemical and clinical criteria. Abnormalities in two or more of the following serum components - potassium, uric acid, or phosphate - exceeding their normal reference ranges as a result of tumor cell lysis are classified as laboratory TLS. When these biochemical abnormalities are accompanied by a serum creatinine level ≥1.5 times the upper limit of normal, cardiac arrhythmias, or seizures, the condition is defined as clinical TLS. In a retrospective study [3], 53% of patients who developed TLS had severe renal dysfunction at diagnosis. Despite supportive therapies, including hydration and rasburicase, the mortality rates remain high, with 63% of patients dying during follow-up [3]. Recent literature highlighted the need for increased awareness and early intervention to improve outcomes as TLS is increasingly recognized in solid tumors owing to advancements in cancer therapies [7]. However, early diagnosis of TLS is challenging as it often presents with nonspecific symptoms. Furthermore, TLS in solid tumors is extremely rare, leading to frequent underestimation of the risk and a lack of preventive measures. In cases of solid tumors with identifiable risk factors for TLS, monitoring the onset of TLS through post-chemotherapy blood tests is crucial. We herein describe a case of TLS associated with chemotherapy for ascending colon cancer. A blood test was conducted three days after the initiation of chemotherapy, which led to prompt diagnosis and timely therapeutic intervention. We report a case of TLS after obtaining written informed consent from the patient.

## Case presentation

**Figure 1:**
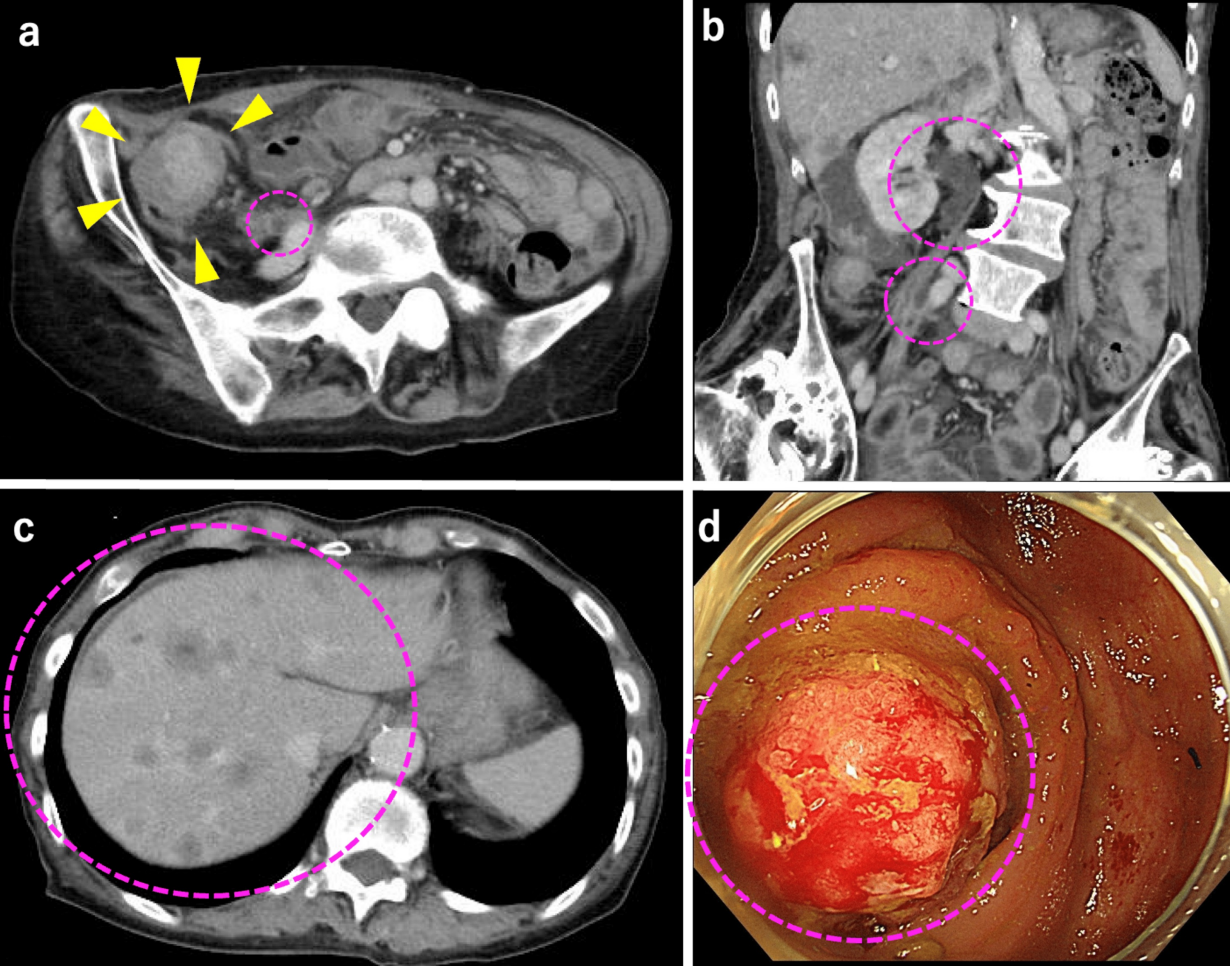
(a, b, c) computed tomography and (d) lower gastrointestinal endoscopy (a, b) Computed tomography (CT) revealed contrast-enhanced wall thickening extending from the cecum to the ascending colon. The right ureter showed contrast-enhanced wall thickening accompanied by hydronephrosis. (c) Multiple hypodense masses of varying sizes in the liver. (d) Lower gastrointestinal endoscopy showed a type 1 tumor in the ascending colon that obstructed the passage of the scope.

The patient was a woman in her 70s. On January 7th, 2023, she presented to our outpatient clinic with a two-week history of diarrhea and vomiting. She was admitted with a diagnosis of ileus caused by ascending colon obstruction. Computed tomography showed contrast-enhanced wall thickening extending from the cecum to the ascending colon, multiple hypodense masses of varying sizes in the liver, and contrast-enhanced thickening of the right ureter, accompanied by hydronephrosis (Figure 1). Lower gastrointestinal endoscopy revealed a type 1 tumor in the ascending colon that obstructed the passage of the scope (Figure 1), which led to the diagnosis of ascending colon cancer accompanied by liver metastasis and ureteral invasion.

**Figure 2:**
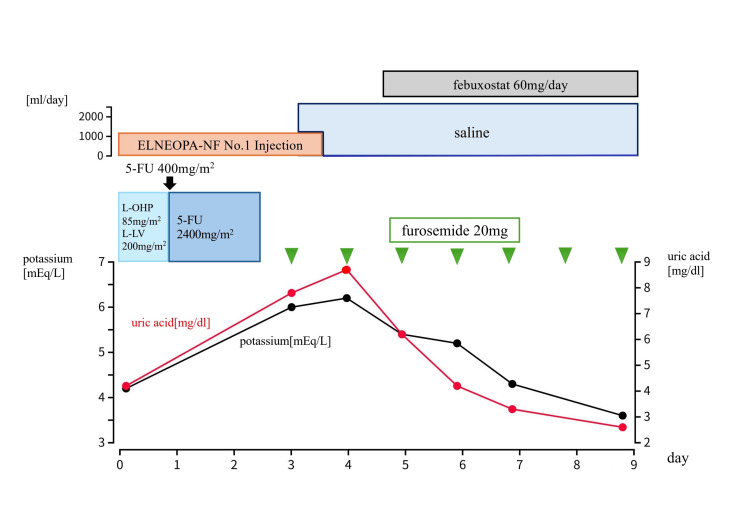
Course of treatment during mFOLFOX6 therapy On day three following the initiation of mFOLFOX6 therapy, tumor lysis syndrome was diagnosed due to elevations in serum uric acid, potassium, and creatinine levels. High-volume intravenous hydration and furosemide (20 mg/day) were initiated immediately after symptom onset to manage hyperkalemia. Febuxostat (60 mg/day) was added on day four in response to further elevation of uric acid, resulting in rapid improvement in laboratory parameters.

On January 24th, a colostomy was performed, and chemotherapy was planned. On February 7th, modified FOLFOX6 (mFOLFOX6) was initiated (Figure 2). The pre-chemotherapy blood tests revealed markedly elevated tumor markers, with CEA at 142.2 ng/mL and CA19-9 at 6013.0 U/mL. Additionally, elevated levels of alkaline phosphatase (ALP; 1198 U/L), lactate dehydrogenase (LDH; 1396 U/L), and C-reactive protein (CRP; 14.58 mg/dL) were also observed. Uric acid, phosphorus, and creatinine were all within normal ranges. On blood tests performed three days after the initiation of chemotherapy, laboratory tests revealed markedly elevated levels of uric acid (7.8 mg/dL) and potassium (6.0 mEq/L), while the creatinine level was only slightly above the upper limit of normal at 1.01 mg/dL.

In accordance with the Cairo-Bishop classification, the patient was diagnosed with laboratory TLS (Table 1). Up to the point of onset, the only physical symptom suggestive of TLS was anorexia on the day of onset, with no other apparent abnormalities observed. Immediately after symptom onset, high-volume rehydration was initiated, and intravenous furosemide (20 mg/day) was administered to manage hyperkalemia. On day four, febuxostat (60 mg/day) was initiated due to the uric acid level further increasing to 8.7 mg/dL, which rapidly improved the laboratory results. On day seven, improvements were observed with a uric acid level of 3.3 mg/dL and a potassium level of 4.3 mEq/L. Regarding serum creatinine, although an elevated level of 1.27 mg/dL persisted on day seven, renal function showed improvement by day nine, with creatinine decreasing to 1.00 mg/dL. Furosemide was discontinued on day eight, and febuxostat was discontinued on day 14. The only observed symptom, loss of appetite, also showed gradual improvement over time in parallel with the normalization of blood test results. Due to reduced performance status during the onset and recovery from TLS and cancer-related fever, as well as the need for rehabilitation, the hospitalization period was prolonged. Subsequently, the patient was discharged on day 28, and the same chemotherapy regimen was continued on an outpatient basis. On day 14 of chemotherapy, the patient developed neutropenia, which prompted a dose reduction of oxaliplatin to 75 mg/m² and discontinuation of bolus administration of 5-FU. In addition, the identification of a positive KRAS G12D mutation introduced bevacizumab from the second cycle. Moreover, from the second cycle, oxaliplatin was administered at 60% of the standard dose, bolus 5-FU was omitted, and infusional 5-FU was administered at 80% of the standard dose, with the addition of bevacizumab (L-OHP at 75 mg/m², l-LV at 200 mg/m², 5-FU at 1920 mg/m² over 46 hours, and bevacizumab at 5 mg/kg). This regimen was repeated up to the 12th cycle. CT after four and nine cycles of therapy revealed reduced and subsequent maintenance of liver metastases. Before the 13th cycle of chemotherapy, cancer progression was not clearly observed. However, persistent hematochezia was observed. The patient declined to undergo lower gastrointestinal endoscopy. The coagulation abnormalities caused by the malignant tumor became more pronounced, and despite blood transfusions, hemorrhage could not be controlled. The patient passed away eight months after treatment initiation. The progression-free and overall survival periods were seven and eight months, respectively.

**Table 1:** Comparison of laboratory parameters from day zero to day nine at FOLFOX chemotherapy

Laboratory parameter	Reference range	Day 0 (Pre-FOLFOX)	Day 3	Day 4	Day 7	Day 9
Uric acid (mg/dL)	2.4 – 7.0	4.2	7.8	8.7	3.3	2.6
Potassium (mEq/L)	3.4 – 4.8	4.2	6	6.2	4.3	3.6
Phosphorus (mg/dL)	2.5 – 4.7	2.7	3.2	4.6	3.7	2.2
Creatinine (mg/dL)	0.60 – 1.10	0.86	1.01	1.24	1.27	1
Lactate dehydrogenase (IU/L)	119 – 229	1396	2537	2230	657	494

## Discussion

In this case, we learned several key lessons regarding TLS. First, TLS has the potential to follow a fatal course. In this case, despite only a mild elevation in serum creatinine, the patient presented with marked hyperkalemia (potassium level of 6.0 mEq/L), placing them at imminent risk for life-threatening arrhythmias. Second, TLS most frequently occurs within a few days following chemotherapy initiation and is often characterized by nonspecific symptoms. For tumors with risk factors for TLS, post-chemotherapy blood tests should be considered.

The incidence of TLS in solid tumors is extremely low, reported to be less than 0.3% [2]. TLS is more commonly observed in hematologic malignancies, and the incidence varies depending on the specific type. Notably, acute lymphoblastic leukemia has been associated with a high incidence rate of approximately 47% [8]. A mortality rate as high as 35% following the onset of TLS in solid tumors has been reported. Compared to hematologic malignancies, the incidence of TLS in solid tumors is significantly lower; however, the high mortality rate may be attributed to delayed diagnosis and treatment due to a lack of clinical awareness and insufficient preventive measures in routine practice.

Several case reports have documented TLS after mFOLFOX6 (5-fluorouracil, leucovorin, and oxaliplatin) therapy in patients with metastatic colorectal cancer (CRC) [9-11]. Of the TLS in the CRC cases, 70% were reportedly associated with therapy, with the median time-to-event being three days and the overall mortality being 60% [12]. In this case, the precise timing of TLS onset was unclear owing to the absence of specific symptoms. Pretreatment blood tests revealed that the UA, potassium, and phosphorus levels were within the normal range, and the patient exhibited no clinical symptoms other than anorexia, suggesting that TLS had not yet developed. On day three following the initiation of chemotherapy, laboratory tests revealed markedly elevated levels of uric acid and potassium; however, serum creatinine was only mildly elevated, and no clinical symptoms such as arrhythmias or seizures were observed. Therefore, the patient was diagnosed with laboratory TLS according to the Cairo-Bishop criteria [6]. Nonetheless, if the condition had not been identified at this point, it could have progressed to clinical TLS without any preceding symptoms. This case underscores the importance of appropriate risk assessment and prophylactic measures at the onset of chemotherapy. 

The approach to TLS prevention in solid tumors varies depending on the patient’s risk classification [13]. Risk assessment for TLS begins with stratification based on disease type, patient age, and tumor burden. Diseases with an estimated incidence of TLS below 1% are categorized as low-risk, those with an incidence of 1-5% as intermediate-risk, and those with an incidence exceeding 5% as high-risk. Renal function is subsequently evaluated using serum creatinine levels to determine the presence of renal impairment, which is then incorporated into the final risk classification, allowing further refinement of low-, intermediate-, or high-risk status. Solid tumors are generally considered to carry a low risk for the development of TLS. However, several factors have been identified that may elevate this risk, including a large tumor burden, presence of liver metastases, elevated lactate dehydrogenase (LDH) or uric acid levels, highly chemosensitive malignancies such as germ cell tumors or small cell lung cancer, impaired renal function, administration of nephrotoxic agents, and concomitant infection or dehydration. Patients with any of these risk factors are classified as intermediate-risk, while those without are considered low-risk. In the low-risk groups, standard fluid hydration and monitoring are sufficient. For intermediate-risk groups, aggressive fluid hydration along with the administration of UA synthesis inhibitors, such as allopurinol or febuxostat, is recommended. For high-risk groups, rasburicase should be considered. Furthermore, diuretics, electrolyte correction, and, if necessary, renal replacement therapy should be considered, in addition to aggressive hydration and hyperuricemia management.

Given the rapid onset of TLS, early detection is imperative. Previous studies have indicated the need to cautiously monitor high-risk patients [13]. Even in patients categorized as low- or moderate-risk groups, regular monitoring of fluid intake and output as well as blood parameters, such as UA, P, K, and Cre, after chemotherapy is necessary, depending on the individual case. Considering that the patient in this case report was diagnosed with colorectal cancer, a type of solid tumor, and had multiple risk factors, and they were classified as having intermediate risk for TLS, thereby requiring the use of UA synthesis inhibitors in addition to hydration therapy. In the present case, prior to the onset of TLS, the only observed symptom was loss of appetite. TLS was first recognized based on elevated levels of potassium, uric acid, and creatinine identified in blood tests performed three days after the initiation of chemotherapy. From day three, during which the diagnosis was established, high-volume hydration was administered, the K level was managed, and diuresis was initiated. On day four, febuxostat was initiated. Despite the presence of hyperuricemia, hyperkalemia, and renal impairment, the treatment proved highly effective, with all the values returning to nearly normal by day seven. The present case can be considered to be an example of TLS development in a patient with ascending colon cancer, classified as an intermediate risk, that improved with prompt diagnosis and treatment. Furthermore, although TLS prevention through chemotherapy dose reduction has been reported in acute leukemia [14], there is currently no evidence supporting this approach for solid tumors. In the present case, the dose was adjusted for subsequent chemotherapy cycles due to the occurrence of neutropenia as an adverse event. However, no dose modification was made specifically for TLS prevention.

As TLS often presents without specific symptoms, making early detection difficult, regular blood tests should be considered for tumors with risk factors for TLS. As of 2019, 11 cases of TLS induced by chemotherapy in patients with CRC aged 15 years and older have been reported. The earliest reported case occurred 18 hours following chemotherapy initiation, and the most common onset was three days after initiation. Numerous TLS cases have been reported in patients with hematologic malignancies; however, only three cases associated with FOLFOX therapy for CRC have been documented in the literature. Similar to this case, the three reported cases successfully recovered (Table 2). Kim et al. reported a patient who developed TLS three days after the initiation of FOLFOX therapy as a second-line treatment, following 12 cycles of FOLFIRI plus bevacizumab (BEV) therapy as a first-line treatment, for descending colon cancer, which was considered to be a progressive disease [9]. This was the first case of TLS associated with mFOLFOX6 therapy. Akansha et al. reported a patient with metastatic CRC who developed TLS one day after the initiation of mFOLFOX6 therapy as a first-line treatment [10]. Furthermore, Gouveia et al. documented a patient with TLS who had left-sided colon cancer and underwent three cycles of mFOLFOX6 therapy; they noted a reduction in liver metastases on CT after the TLS resolution [11]. Although the exact timing of TLS onset after three cycles is not specified in this case report, the other two cases were diagnosed and managed for TLS on days three and one following chemotherapy initiation. In both of these cases, early diagnosis enabled successful management without progression to a fatal outcome. Previous studies have frequently reported the onset of TLS three days after chemotherapy administration. However, this reflects the point at which the condition was diagnosed, and it is highly likely that the actual onset occurred earlier. Therefore, regular blood tests immediately after mFOLFOX6 administration is strongly recommended for patients with CRC who are at risk of developing TLS. Based on previous reports and the present case, it is recommended that patients at high risk for TLS undergo blood testing one to three days after the initiation of chemotherapy [12]. For other chemotherapy regimens and tumor types, the need for post-chemotherapy blood tests should be continuously evaluated as more cases accumulate.

**Table 2:** Summary of TLS in a colorectal cancer patient treated with FOLFOX Comparison between previously reported cases of TLS following FOLFOX therapy in colorectal cancer and the present case TLS - tumor lysis syndrome

Reference	Age/sex	Treatment regimen	Onset timing	Outcome
Kim HD, 2014	59-year-old male	FOLFOX	Day 3	Recovered
Agrawal A, 2016	55-year-old female	FOLFOX	Unknown	Recovered
Gouveia HS, 2018	51-year-old female	FOLFOX	After 3rd course	Recovered
The present case	75-year-old female	FOLFOX	Day 3	Recovered

## Conclusions

We herein report a case of TLS induced by mFOLFOX6 therapy for ascending colon cancer. In this case, blood tests performed on day three after the initiation of chemotherapy enabled the diagnosis of TLS, leading to prompt initiation of treatment. In patients with solid tumors who possess risk factors for the development of TLS, an evaluation for TLS using blood tests should be performed within three days after the initiation of chemotherapy.
